# Neonatal SONography Assisted Resuscitation (NeoSONAR): Scoping review of the current literature and future directions

**DOI:** 10.1038/s41372-025-02458-z

**Published:** 2025-10-29

**Authors:** Yogen Singh, Satyan Lakshminrusimha, Belinda Chan

**Affiliations:** Division of Neonatology, Department of Pediatrics, University of California Davis Children’s Hospital, Sacramento, California USA

**Keywords:** Paediatrics, Translational research

## Abstract

Ultrasound is increasingly utilized during cardiac arrest in adult populations. The European Society of Pediatric and Neonatal Intensive Care and the American Academy of Pediatrics have published guidelines on the use of point-of-care ultrasound (POCUS) in neonates and children. However, there were no specific recommendations for its use during the Neonatal Resuscitation Program (NRP) algorithm. In this narrative review, we map the existing evidence to evaluate its role, potential benefits, and limitations of POCUS integration during neonatal resuscitation. The limited available studies, mostly case reports, suggest that POCUS can aid in the early identification of correctable or modifiable causes, evaluation of cardiac function, detection of heart rate or pulseless electrical activity, and guidance of resuscitative efforts. Significant gaps remain in knowledge, research studying its feasibility and benefits, and a lack of specific training and standardized protocols for integrating POCUS into the NRP algorithm, emphasizing the need for further research.

## Background

Neonatal resuscitation is required in up to 10% of live birth infants. In high-income countries, around 5% of newborns require positive pressure ventilation, 2% require endotracheal intubation, and 0.3% newborns receive chest compressions or emergency medications [1]. Neonatal resuscitation at birth adhering to the Neonatal Resuscitation Program (NRP) is practiced worldwide by trained professionals. It relies predominantly on clinical assessment, which has low sensitivity and specificity for identifying specific underlying causes of perinatal asphyxia [2, 3]. When a newborn fails to respond to initial resuscitation efforts, prompt identification of a reversible or correctable cause is critical for guiding physiology-based specific interventions and improving clinical outcomes.

Point-of-care ultrasound (POCUS) has demonstrated a significant value in cardiac arrest by providing anatomical and hemodynamic information in real-time, thereby improving diagnostic precision and facilitating timely intervention [2, 3], especially in adult advanced life support (ALS) settings. POCUS can help identify treatable causes such as cardiac tamponade, acute pulmonary embolism, myocardial infarction, aortic dissection, and hypovolemia. There is no surprise that it is routinely used in adult emergency care, and there are standardized protocols for its integration in clinical practice. Reynolds et al. (2022) reported that POCUS could detect treatable causes of cardiac arrest with a sensitivity and specificity of 70–90% [2–4]. In another recent prospective trial, Breitkreutz and colleagues reported improved survival rates when ultrasound was used to identify correctable underlying causes and it guided changes in management of cardiac arrest in the adults [5].

Despite these promising findings, the key challenges in using POCUS during ALS resuscitation include operator-dependent variability, the requirement for brief pauses in cardiopulmonary resuscitation (CPR) to acquire diagnostic images, and concerns regarding disruption of the resuscitation workflow. The overall body of evidence to address these challenges remains limited and of low certainty, primarily due to the predominance of observational study designs and the presence of potential biases. The routine use of POCUS during adult resuscitation is approached with caution. The International Liaison Committee on Resuscitation (ILCOR, 2022) recommends the use of POCUS during ALS resuscitation when POCUS-trained providers are available and only if its use does not delay or interfere with chest compressions [5, 6]. More rigorous studies on POCUS-assisted resuscitation are needed, along with adequate training for clinicians and evidence-based protocols for using this promising bedside tool.

There has been a significant interest in use of POCUS in the neonatal intensive care units (NICU). Despite its rapidly growing interest, POCUS-assisted resuscitation in neonates remains significantly under-researched in comparison to its role in adults. Both the American Academy of Pediatrics (AAP) and the European Society of Pediatric and Neonatal Intensive Care (ESPNIC) have endorsed the use of POCUS for diagnostic and procedural purposes, including the identification of treatable causes in a collapsing infant that may lead to cardiac arrest [7, 8]. However, despite these endorsements and the recognized potential benefits, the integration of POCUS into neonatal and pediatric resuscitation algorithms remains largely unexplored. This narrative review summarizes the current use of ultrasound during cardiac arrest in children, with a special focus on its use during neonatal resuscitation.

## Materials and methods

The objective of this narrative review was to map and synthesize the existing literature on the use of POCUS during cardiac arrest in neonates and children. Specifically, we aimed to identify the reported applications, benefits, and limitations of POCUS in this context.

We conducted a comprehensive literature search using the PubMed database, applying the following search terms: “Cardiopulmonary Resuscitation,” “chest compression,” “basic life support,” “Heart Arrest/diagnostic imaging,” “Heart Massage,” “cardiac massage,” “Ultrasonography,” and “Echocardiography.” The search was limited to English-language articles published between 2015 and 2024.

### Inclusion criteria were as follows

- Studies involving the use of ultrasound during cardiac arrest or active chest compressions
- Studies reporting ultrasound applications (e.g., diagnostic or procedural), indications (e.g., evaluation for reversible causes), or clinical outcomes (e.g., return of spontaneous circulation [ROSC], survival, diagnostic yield)
- Studies involving neonates ( < 28 days old) or children (1 month to 17 years old)
- Full-text articles, including randomized controlled trials, prospective studies, cohort studies, and case reports

### Exclusion criteria included

- Studies not involving cardiac arrest
- Studies conducted in non-human subjects
- Simulation studies without clinical data (unless directly relevant)
- Studies in which ultrasound was used only outside of the acute resuscitation phase (e.g., post-arrest monitoring)
- Reviews, expert opinions, guidelines without patient data, and abstracts without full-text availability

We manually screened the reference lists of included studies to identify additional relevant publications.

Following the screening and eligibility assessment, 13 studies were identified that met the inclusion criteria and were most pertinent to the neonatal and pediatric populations. Data were extracted on the study design, patient population, ultrasound applications, clinical outcomes, and reported limitations. The findings were synthesized narratively, with a primary focus on neonatal applications.

## Results

Thirteen studies on use of ultrasound during neonatal and pediatric resuscitation were identified (Table 1), spanning various clinical settings—including delivery rooms (DR), neonatal intensive care units (NICUs), pediatric emergency departments (PEDs), and pediatric intensive care units (PICUs). These studies involved patients ranging from neonates ( < 28 days) and infants ( < 1 year) to children up to 17 years of age. Among these studies, only 51 neonates were explicitly mentioned to have received ultrasound scanning during resuscitation in the NICU or delivery unit [9–14]. Most of the available literature consists of case reports as summarized in Table 1. We have focused our results and discussion on sonographic assessment during resuscitation in neonates and children.

**Table 1:** Summary of published literature on use of ultrasound during resuscitation of neonates and children.

Reference #	Author(s), Year	Study Design	Age Range (<1mon, 1mon-17 years, > 18 years old)	# of neonate (<28 days old)	Setting	Types of POCUS Performed	Indications	Outcomes/Key Findings
[9]	Rodriguez-Fanjul J et al.	Case Report	<1	2	DR, NICU	Cardiac, Lung	CA in newborns	POCUS diagnosed hypovolemia and pneumothorax as the reversible causes of CA.
[10]	Ibarra-Ríos D et al., 2023	Observational	<1	25	DR, NICU	Cardiac, Lung	CA, hemodynamic deterioration	25 POCUS scans using modified SAFE-R protocol were performed on neonates with acute decompensation. Five life-threatening cases resolved after identifying and treating reversible causes.
[11]	Chan B et al.	Case Report	<1	1	DR, NICU	Cardiac	CA	Cardiac POCUS revealed that a newborn presumed to have PEA exhibited cardiac contractility but was insufficient to generate effective cardiac output, consistent with pseudo-PEA.
[12]	Polito A et al.	Case Report	<1	1	Cardiac OR	Lung	CA, PEA	Lung POCUS diagnosed tension pneumothorax as the cause of CA. ROSC after chest tube placement with a total of 30 min of resuscitation.
[13]	Azzopardi E et al.	Case Report	1–17	0	Cardiac OR	Cardiac	CA	Cardiac POCUS demonstrated poor cardiac contractility that responded to inotropes rather than more fluid bolus in one patient. Another two cases showed cardiac standstill without reversible causes and stopped resuscitation.
[14]	Morgan RW et al.	Case Report	1–17	0	PICU	Cardiac	CA, PEA	POCUS diagnosed pulmonary emboli as the cause of CA.
[15]	Moher JM et al.	Retrospective	1–17	0	PED	Cardiac, Lung	Respiratory distress, CA	Of 225 POCUS scans performed on 142 pediatric ED patients, 8% were for shock or arrest. Cardiac scans showed abnormalities (reduced function, pericardial effusion, or structural defects) in 69.1%, though arrest-specific findings were not detailed. Among 35 lung scans, five pneumothoraxes were identified.
[16]	Leviter JI et al., 2023	Case Report	<1, 1-17	0	PED	Cardiac, Femoral artery	CA	POCUS was used to assess cardiac activity, detect femoral pulses, and confirm intraosseous catheter was functional properly.
[17]	Lalande E et al.	Systematic Review and Meta-Analysis	All ages	0	Out-of-hospital, ED	Cardiac	Blunt and penetrating traumatic CA	Patients without cardiac activity on cardiac POCUS was unlikely to survive, but only 2 pediatric cases were included in all 8 studies.
[18]	Steffen K et al.	Case Report	<1, 1-17	1	PED, NICU, PICU	Cardiac	CA	Three cases of pediatric patients, 1 neonate, with cardiac standstill achieved ROSC. Cardiac standstill was less predictive of death unlike in adults.
[19]	Kayama K et al.	Prospective	1–17	21	DR, NICU	Cardiac	Assess accuracy of detecting HR using POCUS in normal and neonatal arrest or asphyxia.	Cardiac doppler could detect HR within 10 s in 18 out of 21 neonatal resuscitation cases, with five neonates had bradycardia with 1-min APGAR < 6. HR was displayed with a median time of 5 s.
[20]	Leviter JI et al.	Prospective	1–17	0	PED	Cardiac, Femoral artery	Evaluate feasibility of obtaining optimal images in 10 s	On 22 pediatric stable patients, 22 experienced sonographers obtained interpretable cardiac views in 86% (apical four-chamber), 94% (subxiphoid), and 75% (femoral artery) of cases within the 10s-time limit.
[21]	Yanni E et al.	Survey	1–17	0	PED	Cardiac	Evaluate inter-rate agreement on cardiac standstill ultrasound video clips	110 experienced PED attendings reviewed 11 cardiac POCUS videos obtained during CA in pediatric patients. Agreement of cardiac standstill diagnosis was acceptable when wall motion matched valve motion but unacceptable when wall motion occurred without valve motion.

*CA* Cardiac arrest, *DR* Delivery room, *HR* Heart rate, *NICU* Neonatal intensive care unit, *OR* Operating room, *PED* Pediatrics emergency department, *PICU* Pediatrics intensive care unit, *POCUS* Point of care ultrasound, *PEA* Pulseless electric activity, *ROSC* Return of spontaneous circulation, *S* Seconds, and # - Number.

### Role of POCUS during neonatal and pediatric cardiac arrest

**Fig. 1:**
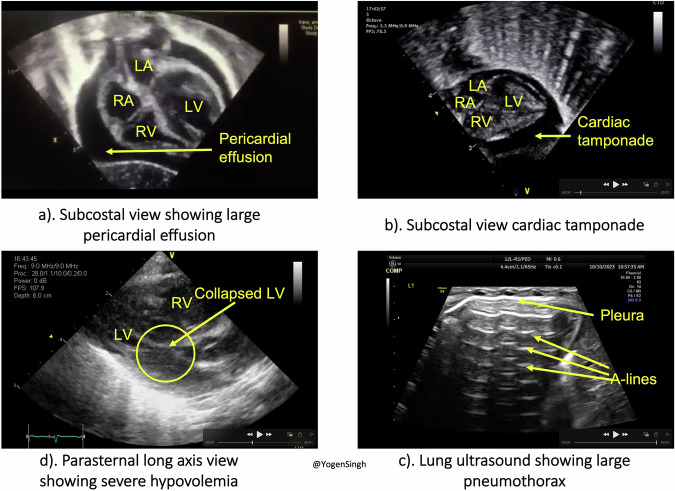
Ultrasound images acquired during neonatal resuscitation. Image **a** subcostal view showing large pericardial effusion, Image **b** subcostal view showing collapsed right atrium and right ventricle with pericardial effusion consistent with cardiac tamponade needing neonatal resuscitation – image acquired during chest compressions and then ultrasound guided pericardiocentesis performed, Image **c** showing a large pneumothorax with no lung sliding of pleura, excessive A-lines evenly spaced horizontal echogenic lines below the pleura) and no B-line (vertical echogenic lines extending below the pleura), and Image **d** showing a parasternal long axis view showing severe hypovolemia – “kissing sign” of left ventricle (collapsed left ventricle cavity).

POCUS was used primarily for lung and cardiac assessments to identify reversible or correctable causes of arrest such as pneumothorax, pericardial effusion, decreased contractility, hypovolemia, and pulmonary embolism [9–16]. Only three case reports described the use of ultrasound specifically during birth resuscitation in the delivery room or in the NICU [9–11]. Other case reports or literature involved older infants and children in different emergency settings [11–15]. Lung POCUS enabled rapid detection of pneumothorax, prompting emergent thoracentesis and resulting in restoration of effective oxygenation, ventilation, and heart rate [9, 12, 15, 16]. Cardiac POCUS was reported to guide clinical decisions, such as administering inotropes instead of fluid boluses when poor contractility was observed [10, 11, 13, 16] (Fig. 1).

In four pediatric cases of cardiac arrest, pulmonary emboli were identified based on the venous thromboembolism risk factors and bedside echocardiography. Early accurate diagnosis on sonographic assessment helped in early administration of intravenous tissue plasminogen activator during CPR or shortly after ROSC [14].

Cardiac POCUS also facilitated the diagnosis of pseudo-PEA in one neonate, where cardiac contraction was found on ultrasound, but it was insufficient to generate adequate cardiac output to support tissue perfusion or peripheral arterial pulsatility [11]. As pseudo-PEA progressed to cardiac standstill, use of POCUS during resuscitation provided critical information that guided decisions to terminate resuscitation [12, 17]. Notably, unlike in adults—where cardiac standstill strongly predicts mortality—pediatric patients have occasionally achieved return of spontaneous circulation (ROSC) when supported with extracorporeal membrane oxygenation (ECMO) and restoration of coronary perfusion [18].

Leviter et al. reported the use of ultrasound to detect femoral pulses in the pediatric emergency department [16]. Color Doppler ultrasound was used to assess heart rate and confirm pulses during resuscitation. Similarly, Kayama and colleagues conducted a prospective study evaluating the use of a fetal Doppler probe placed over the chest to detect heart rate in newborn. Although the device does not generate a visual ultrasound image, it uses sonographic technology and was applied in real-time clinical settings, thus meeting inclusion criteria for our review. The study found that the Doppler probe detected heart rate significantly faster than electrocardiography (median 5 vs. 10 s, *p* < 0.001). It successfully identified heart rate within 10 s in 18 out of 21 resuscitations, including five cases of bradycardia (heart rate <100 bpm) [19].

In addition to diagnostic applications, ultrasound can aid with procedural success and confirm umbilical line placement [7, 8]. However, none of the included studies reported its use for these purposes during active resuscitation, except for one case in which ultrasound confirmed proper intraosseous catheter function [16].

### Feasibility of POCUS during neonatal and pediatric cardiac arrest

Leviter and colleagues evaluated the feasibility of imaging acquisition within these time constraints in patients under 12 years old. They found that experienced sonographers were able to obtain optimally interpretable cardiac views in 86% of apical four-chamber, 94% of subxiphoid, and 75% of femoral artery scans within the agreed time limit [20].

Yanni et al. assessed interrater reliability in interpreting 11 cardiac POCUS videos obtained during pediatric cardiac arrest. Among 110 experienced pediatric emergency physicians, the interrater agreement was acceptable when wall motion matched valve motion (κ = 0.740; 95% CI, 0.735–0.745). However, agreement dropped to unacceptable levels when wall motion was present without associated valve movement (κ = 0.304; 95% CI, 0.287–0.321) [21].

### Integrating POCUS into resuscitation workflow

In our research, there is paucity of data on examining integration of ultrasound during resuscitation of neonates and children. Rodriguez-Fanjul et al. and Chan et al., as POCUS experts, recommend performing POCUS during pulse checks, particularly after a poor response to the initial dose of epinephrine [9, 12]. Ibarra-Rios et al. further modified the Sonographic Assessment of liFe-threatening Emergencies – Revised (SAFE-R) protocol and reported its clinical utility - 5 out of 25 neonates assessed with the modified protocol had life-threatening conditions identified and successfully treated with targeted interventions [10].

## Discussion

Our literature review identified 13 published studies describing the use of POCUS in 51 neonates undergoing resuscitation in the NICU or in the delivery unit, applied across a variety of clinical settings and indications. Based on the authors’ anecdotal experience, it is very likely that additional cases exist but remain unpublished or are not captured in the current literature. To date, no prospective studies or systematic approaches have specifically evaluated the integration of POCUS into neonatal or pediatric resuscitation.

A standardized, protocol-driven approach may improve the effectiveness of POCUS-guided resuscitation, as demonstrated in the adult population. Several well-established adult scanning protocols and guidelines have been published, including Rapid Ultrasound in SHock (RUSH), Focused Assessment with Sonography for Trauma (FAST), the Extended FAST (EFAST), and the Bedside Lung Ultrasound in Emergency (BLUE) protocol—all of which outline systematic scanning sequences [2, 22–26]. The POCUS-CA protocol is specifically designed for use during adult cardiac arrest.³ Notably, one study has also validated the RUSH protocol in pediatric patients with shock and hypotension [26]. Similar structured scanning sequences have been adapted for critically ill or decompensating neonates, including the SAFE-R protocol and the Crashing Neonatal Protocol (CNP), but there is no published body of evidence on the integration of POCUS during the NRP algorithm [27, 28]. While these structured POCUS protocols provide guidance on sonographic assessment of critically ill or decompensating infants, these frameworks have not yet been validated for feasibility, diagnostic accuracy, or clinical impact during neonatal resuscitation in the delivery unit.

Despite these advances and promising results from small studies or case reports, POCUS is still not routinely incorporated into resuscitation efforts in adults, largely due to concerns about interrupting chest compressions and the limited availability of trained providers capable of acquiring and interpreting images rapidly [4]. These barriers are even more pronounced in neonatal resuscitation, where there are not even feasibility studies assessing POCUS assessment within the NRP framework.

To the best of our knowledge, no standardized protocols specifically addressing the use of POCUS during neonatal resuscitation. Its feasibility, efficacy, and diagnostic accuracy in this context remain unproven. The key challenges include lack of evidence providing confidence to the clinicians, lack of trained personnel, lack of training structure and equipment, concerns regarding potential delays in chest compressions, interference with NRP workflow, and the risk of poor image quality leading to misdiagnosis during time-critical assessments. These limitations and knowledge gaps need addressing through high-quality research before POCUS can be safely and effectively adopted for routine use—even by experienced practitioners.

**Fig. 2:**
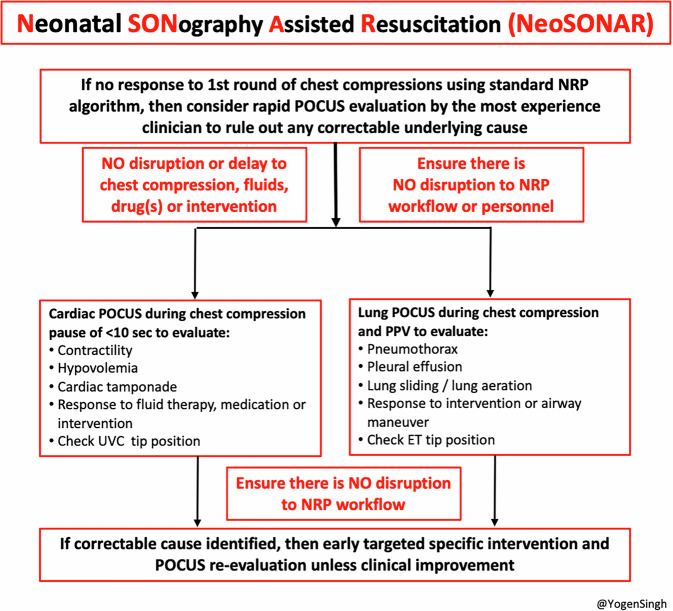
Neonatal SONographic Assisted Resuscitation (NeoSONAR). This figure illustrates how targeted use of POCUS can be applied during neonatal resuscitation using NRP algorithm, especially in infants who do not respond to standard measures.

**Fig. 3:**
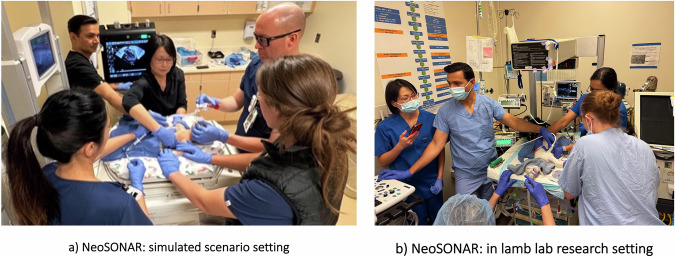
Application of ultrasound during neonatal resuscitations. Image **a** in a simulated scenario teaching setting, and Image **b** real-time scanning during lamb resuscitation using Neonatal SONographic Assisted Resuscitation (NeoSONAR) in research setting.

As a first step toward addressing these gaps, we propose a structured approach to integrate POCUS into resuscitation in neonates who do not respond to standard NRP protocol, and without interrupting workflow or compromising the quality of chest compressions: the Neonatal SONography Assisted Resuscitation (NeoSONAR) protocol. Figure 2 and Fig. 3 demonstrated when, what stage, and how NeoSONAR can be introduced during neonatal resuscitation (Figs. 2 and 3).

**Fig. 4:**
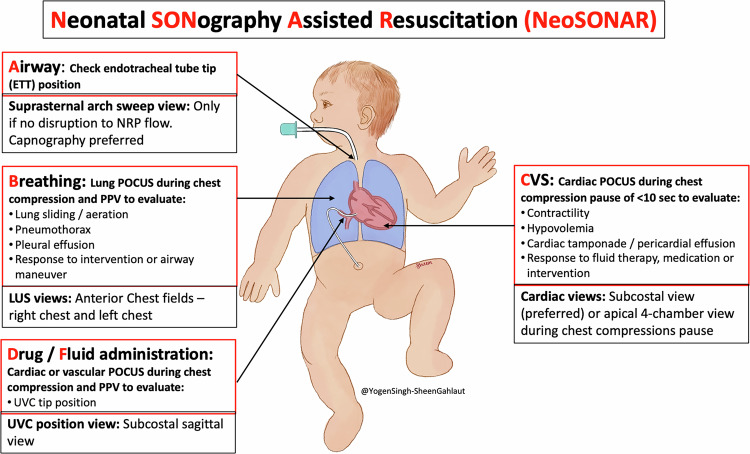
A simple NeoSONAR protocol on the potential use of ultrasound during neonatal resuscitation and guide management. This simple protocol with A-D steps provides guidance for the emergency POCUS indications and simple views which can aid in rapid assessment and target-specific intervention.

**Fig. 5:**
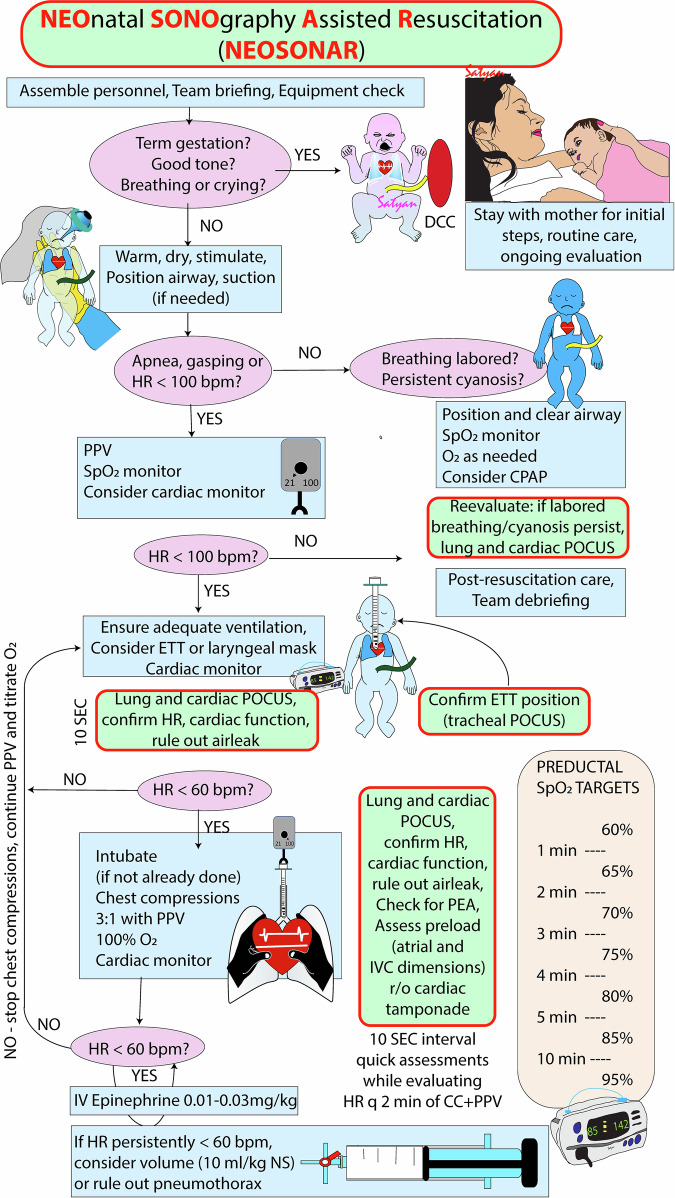
Integration of ultrasound assessment with the Neonatal Resuscitation Protocol (NRP) illustrating POCUS’s role and value in the perinatal period.

Recent studies show that a large proportion of neonatal care providers use POCUS in their clinical practice, and the use of sonographic assessment in neonates is exponentially increasing [29–32]. Increasing numbers of neonatologists have become trained in POCUS and targeted neonatal echocardiography. When a POCUS-trained clinician is present, then POCUS could integrate into the NRP algorithm and adjunct resuscitation efforts as illustrated in Figs. 4 and 5 (below).

## Conclusion

POCUS is a valuable tool during cardiac arrest, offering the potential for early diagnosis, identification of reversible causes, and guidance of resuscitation efforts. However, there is an urgent need for systematic evaluation of its feasibility, efficacy, and diagnostic accuracy in neonatal resuscitation through rigorous research. Developing and validating a standardized protocol, such as NeoSONAR, will be essential for safely integrating POCUS into resuscitation workflows. Rigorous research is needed before POCUS can be adopted as a routine component of neonatal resuscitation practice.
